# Multidrug Resistance and Molecular Characterization of *Streptococcus agalactiae* Isolates From Dairy Cattle With Mastitis

**DOI:** 10.3389/fcimb.2021.647324

**Published:** 2021-04-30

**Authors:** Luciana Hernandez, Enriqueta Bottini, Jimena Cadona, Claudio Cacciato, Cristina Monteavaro, Ana Bustamante, Andrea Mariel Sanso

**Affiliations:** ^1^ Laboratorio de Inmunoquímica y Biotecnología, CIVETAN (CONICET), Facultad de Ciencias Veterinarias, Universidad Nacional del Centro de la Provincia de Buenos Aires (UNCPBA), Tandil, Argentina; ^2^ Laboratorio de Microbiología Clínica y Experimental, CIVETAN (CONICET), Facultad de Ciencias Veterinarias, Universidad Nacional del Centro de la Provincia de Buenos Aires (UNCPBA), Tandil, Argentina

**Keywords:** *Streptococcus agalactiae*, virulence, dairy cattle mastitis, multidrug resistance, serotypes

## Abstract

*Streptococcus agalactiae* is a pathogen-associated to bovine mastitis, a health disorder responsible for significant economic losses in the dairy industry. Antimicrobial therapy remains the main strategy for the control of this bacterium in dairy herds and human In order to get insight on molecular characteristics of *S. agalactiae* strains circulating among Argentinean cattle with mastitis, we received 1500 samples from 56 dairy farms between 2016 and 2019. We recovered 56 *S. agalactiae* isolates and characterized them in relation to serotypes, virulence genes, and antimicrobial susceptibility. Serotypes III and II were the most prevalent ones (46% and 41%, respectively), followed by Ia (7%). In relation to the 13 virulence genes screened in this study, the genes *spb1, hylB, cylE*, and PI-2b were present in all the isolates, meanwhile, *bca*, *cpsA*, and *rib* were detected in different frequencies, 36%, 96%, and 59%, respectively. On the other hand, *bac, hvgA, lmb*, PI-1, PI-2a, and *scpB* genes could not be detected in any of the isolates. Disk diffusion method against a panel of eight antimicrobial agents showed an important number of strains resistant simultaneously to five antibiotics. We also detected several resistance-encoding genes, *tet(M), tet(O), ermB, aphA3*, and *lnu(B)* (9%, 50%, 32%, 32%, and 5%, respectively). The results here presented are the first molecular data on *S. agalactiae* isolates causing bovine mastitis in Argentina and provide a foundation for the development of diagnostic, prophylactic, and therapeutic methods, including the perspective of a vaccine.

## Introduction

*Streptococcus agalactiae*, or group B Streptococcus (GBS), was first described from bovines (Nocard and Mollereau, 1887) and for seven decades was exclusively associated with mastitis in dairy herds. Later emerged as a leading cause of human neonatal infections (Manning, 2014), and nowadays *S. agalactiae* is increasingly recognized as an adult invasive pathogen worldwide (Skoff et al., 2009). Also, *S. agalactiae* infections have been reported in many fish species, particularly is an emerging pathogen in Nile tilapia (*Oreochromis nilo*ticus) worldwide (Mian et al., 2009). Recent studies debate if this zoonotic potential remains nowadays and suggest that although *S. agalactiae* is well adapted to various hosts, interspecies transmission is possible and occurs (Morach et al., 2018). Further, they hypothesize about possible routes through which this bacterium could be transmitted between cattle and humans (Botelho et al., 2018).

According to their epidemiology, mastitis pathogens can be classified as contagious or environmental. Contagious pathogens are those for which the udders of infected cattle act as the main reservoir (Cervinkova et al., 2013). *S. agalactiae* has been recognized as a highly contagious obligate parasite of the bovine mammary gland, which generally does not survive for long periods outside of the mammary gland (Keefe, 1997). However, at present, it was demonstrated that the bacteria can survive in extramammary sources (Cobo-Ángel et al., 2018). In cattle, intramammary infections are usually chronic and subclinical, with intermittent episodes of clinical mastitis. Bovine mastitis is the dominant health disorder leading to diminished milk quality and production and is responsible for significant economic losses in the dairy industry (Zadoks et al., 2004; Hogeveen et al., 2011).

Despite numerous eradication programs in cattle, *S. agalactiae* remains a common cause of infections, with high levels of prevalence and contagion in dairy herds in different geographical areas, particularly, in South America countries (Keefe, 2012). In studies conducted in dairy herds of Colombia, the prevalence of *S. agalactiae* varied from 28 to 35% (Ramírez et al., 2014; Reyes et al., 2015); in Brazil, a research group reported this pathogen as the third most prevalent bacteria causing bovine mastitis (Tomazi et al., 2018).

In Argentina, there is no updated data at the national level, but only for some particular regions, such as Córdoba province (Dieser et al., 2014), or other collected between 1999 and 2007, in a larger region that report an *S. agalactiae* prevalence of 29% among cows with mastitis from Buenos Aires, Santa Fe and Córdoba (Calvinho, 2017). A bacteriological study of dairy farms located in the Cuenca Mar y Sierras (Buenos Aires province), one of the main dairy regions of the country, showed *S. agalactiae* to be a frequent etiological pathogen causing subclinical mastitis (Amand De Mendieta et al., 2001). Furthermore, 10% of the sampled dairy farms in this area were positive to this species (Bottini, personal communication).

The pathogenesis of *S. agalactiae* infection and the severity of the disease have been related to the presence of a series of virulence factors mainly involved in colonization of the host, in the dissemination of the bacteria, the evasion of the immune response and internalization in the mammary gland cells. One of the most important factors involved in virulence is the capsular polysaccharide (Cps) (Slotved et al., 2007). *S. agalactiae* can be classified into 10 serotypes according to the type of Cps (Ia, Ib, and II to IX).

Antimicrobial therapy remains the main strategy for the control of this bacterium in dairy herds and human infections. Antimicrobial resistance is an area of concern in both human and veterinary medicine (Schwarz et al., 2010). Currently, there is little official information on the use of different antibiotics in veterinary medicine and, therefore, on the resistance models of animal pathogens circulating in the world. Strain characterization and surveillance are important to obtain information that allows evaluating the level and evolution of antimicrobial resistance (OIE - World Organization for Animal Health, 2018). For this reason, and in particular, studies on the antimicrobial activity of mastitis pathogens are necessary for controlling induced resistance and obtaining useful information for therapeutic decisions (Denamiel et al., 2005).

In this study, we present the first molecular data on *S. agalactiae* isolates causing bovine mastitis in Argentina and provide information in relation to serotypes, virulence, and antimicrobial susceptibility.

## Materials and Methods

### Isolation of *S. agalactiae*

A total of 1500 samples recovered from different cows presenting clinical or subclinical mastitis, except one obtained from a milk tank (B3), between December 2016 and August 2019, were received at the lab. Samples came from 56 dairy farms (herds) located in one of the largest milk-producing regions of Argentina, the Cuenca Mar y Sierras, and *S. agalactiae* isolates could be obtained from seven dairy farms (A, B, C, D, E, G, and H).

Milk samples were collected under aseptic conditions from cows affected by clinical or subclinical mastitis, immediately refrigerated at 4°C and subjected to bacteriological analysis within 24 h of collection. A loopful of milk sample was streaked on trypticase soy agar (TSA) enriched with 5% bovine blood and plates were incubated at 37°C in atmosphere with 5% CO_2_. Subsequently, the plates were examined for colony morphology, pigmentation and hemolytic characteristics after 24–48 h. Presumptive colonies of *Streptococcus* species were selected and streaked into a slant agar for 24 h for biochemical tests, and Gram staining. Catalase, NaCl, bile-esculin, Christie-Atkins-Munch-Peterson (CAMP), hippurate hydrolysis, and sorbitol tests were carried out as described by the National Mastitis Council (2017). Sixty-eight isolates were identified as *S. agalactiae*, being Gram-positive cocci, CAMP reaction-positive, and catalase and esculin activity-negative, and stored at −20°C.

### Molecular Characterization

The DNA template was obtained by boiling bacterial colonies suspended in sterile water for 10 min. To confirm the species identification, a region of the monocopy regulatory gene *dltR*, specific to *S. agalactiae*, was amplified (Lamy et al., 2006). The capsular type identification, Ia, Ib, II-IX, was determined by PCR according to Imperi et al. (2010). Additionally, a total of ten virulence genes, *bac, bca cpsA*, *cylE, hvgA*, *hylB, lmb, rib, scpB*, *spb1*, plus three pili genes designated as pilus island 1, PI-1, PI-2a, and PI-2b, were detected according to previous studies. The examined virulence genes have been associated with adhesion and colonization, invasion, tissue damage, and/or immune evasion (**Table 1**). The PCR products were visualized in 2% agarose gel stained by ethidium bromide.

**Table 1:** *Streptococcus agalactiae* virulence genes assessed by PCR in the present study.

Gene	Encoded protein/function	Reference for primers and PCR conditions
*bac*	surface protein ß-C	Smith et al. (2007)
*bca*	surface protein ą-C	Smith et al. (2007)
*cpsA*	capsular polysaccharide	Bidet et al. (2003)
*cylE*	β-hemolysin	Otaguiri et al. (2013)
*hvgA*	hypervirulent GBS adhesin	Lamy et al. (2006)
*hylB*	hyaluronidase	Otaguiri et al. (2013)
*lmb*	laminin-binding protein	Duarte et al. (2005)
*rib*	surface protein	Smith et al. (2007)
*scpB*	C5a peptidase	Bidet et al. (2003)
*spb1*	surface protein	Smith et al. (2007)
PI-1, PI-2a PI-2b	pilus structures	Martins et al. (2010)

### Data Analysis

Taking into account the combinations of the genes detected in the present study, the virulence profiles were defined. A cluster analysis was carried out using the UPGMA clustering method. The dendrogram was generated using the BioNumerics v.6.6 software.

### Antimicrobial Susceptibility

#### Antimicrobial Susceptibility Testing

The isolates were tested for susceptibility to eight antibiotics using a disc diffusion method according to the CLSI - Clinical and Laboratory Standards Institute (2019) instructions. The antimicrobial agents were selected taking into account their use for mastitis treatment in cattle (penicillin, oxacillin, kanamycin, pirlimycin, and tetracycline) or/and in human medicine (penicillin, erythromycin, clindamycin, levofloxacin). A bacterial suspension in sterile saline solution from an overnight pure culture, adjusted to a turbidity of 0.5 on the McFarland scale, was inoculated on a Muller-Hinton agar (Britania) plate, supplemented with 5% sheep blood. Antibiotic discs (Britania) were placed on the agar surface and plates were incubated overnight (16–18 h) at 37°C in atmosphere with 5% CO_2_. The diameters of the zones of inhibition were then measured and data were interpreted by using human breakpoints values for all the antimicrobial agents except for pirlimycin, for which veterinary interpretive criteria for cattle were available (CLSI - Clinical and Laboratory Standards Institute, 2018; CLSI - Clinical and Laboratory Standards Institute, 2019) (**Table S1**). In relation to the aminoglycoside kanamycin, we used high load antibiotic discs in order to predict lack of synergy when associated with Beta-lactams. Since not kanamycin standards are available, only isolates presenting no zones of inhibition were considered as resistant. The following discs were used: clindamycin (2 μg), pirlimycin (2 μg), erythromycin (15 μg), levofloxacin (5 μg), penicillin (10 units), oxacillin (1 μg), tetracycline (30 μg), and kanamycin (120 μg). Isolates showing resistance against three or more different classes of antibiotics were defined as multi-drug resistant (MDR) (Sweeney et al., 2018).

### Detection of Antimicrobial Resistance Genes

The macrolide resistance gene *ermB* was amplified by PCR according to Zhou et al. (2011), *mef*A, and tetracycline resistance genes *tet*(M), *tet*(O), *tet*(T), and *tet*(K), according to Lopardo et al. (2003), lincosamide resistance gene *lnu*(B) (before named *linB*), according to Bozdogan et al. (1999) and, aminoglycosides resistance genes *aphA3* and *aad6*, according to Poyart et al. (2003). The correlation between phenotype of resistance and resistance genes was analyzed as well as was done by Liu et al. (2017) and Tian et al. (2019).

## Results

Out of 1500 samples received during 33 months from 56 dairy farms located in the Cuenca Mar y Sierras, Buenos Aires Province, Argentina, 68 *Streptococcus agalactiae* strains were identified biochemically. Then, among the original 68 isolates, species-specific PCR (*dltR* gene amplification) confirmed 56 *Streptococcus agalactiae* strains arising from seven dairy farms.

The bovine isolates studied here belonged to capsular genotypes Ia, II, III, and three ones were designed as non-typeable (NT), according to the multiplex PCR. Overall, type III and II were the most prevalent accounting for 26 isolates (46%) and 23 isolates (41%), respectively and, type Ia by 4 isolates (7%) (**Table 2**).

**Table 2:** Distribution of virulence and antimicrobial resistance genes detected among *Streptococcus agalactiae* isolates recovered from dairy cattle with mastitis in Argentina.

			Virulence genes							Antimicrobial resistance genes				
Source	Serotype	Number of isolates	*bca*	*cpsA*	*cylE*	*hylB*	PI-2b	*rib*	*spb1*	*tet* (M)	*tet*(O)	*ermB*	*aphA3*	*lnu* (B)
A	Ia	1	–	–	1 (2%)	1 (2%)	1 (2%)	–	1 (2%)	–	1 (2%)	1 (2%)	–	–
	II	5	1 (2%)	5 (9%)	5 (9%)	5 (9%)	5 (9%)	4 (7%)	5 (9%)	–	4 (7%)	4 (7%)	1 (2%)	1 (2%)
	III	20	–	20 (36%)	20 (36%)	20 (36%)	20 (36%)	20 (36%)	20 (36%)	–	7 (13%)	–	6 (11%)	–
	NT	1	–	–	1 (2%)	1 (2%)	1 (2%)	–	1 (2%)	–	1 (2%)	1 (2%)	–	–
B	Ia	1	–	1 (2%)	1 (2%)	1 (2%)	1 (2%)	–	1 (2%)	–	1 (2%)	1 (2%)	–	–
	II	9	1 (2%)	9 (16%)	9 (16%)	9 (16%)	9 (16%)	3 (5%)	9 (16%)	–	9 (16%)	9 (16%)	2 (4%)	2 (4%)
	III	1	1 (2%)	1 (2%)	1 (2%)	1 (2%)	1 (2%)	1 (2%)	1 (2%)	–	1 (2%)	1 (2%)	1 (2%)	–
	NT	1	–	1 (2%)	1 (2%)	1 (2%)	1 (2%)	–	1 (2%)	–	1 (2%)	1 (2%)	1 (2%)	–
C	Ia	1	1 (2%)	1 (2%)	1 (2%)	1 (2%)	1 (2%)	–	1 (2%)	1 (2%)	1 (2%)	–	1 (2%)	
D	II	7	7 (13%)	7 (13%)	7 (13%)	7 (13%)	7 (13%)	–	7 (13%)	1 (2%)	1 (2%)	–	1 (2%)	–
E	II	2	2 (4%)	2 (4%)	2 (4%)	2 (4%)	2 (4%)	–	2 (4%)	–	–	–	–	–
	NT	1	1 (2%)	1 (2%)	1 (2%)	1 (2%)	1 (2%)	–	1 (2%)	–	–	–	–	–
G	III	5	5 (9%)	5 (9%)	5 (9%)	5 (9%)	5 (9%)	5 (9%)	5 (9%)	3 (5%)	–	–	5 (9%)	–
H	Ia	1	1 (2%)	1 (2%)	1 (2%)	1 (2%)	1 (2%)	–	1 (2%)	–	–	–	–	–
**Total**		**56**	**20 (36%)**	**54 (96%)**	**56 (100%)**	**56 (100%)**	**56 (100%)**	**33 (59%)**	**56 (100%)**	**5 (9%)**	**27 (48%)**	**18 (32%)**	**18 (32%)**	**3 (6%)**

The virulence genes *bac*, *scpB* and *lmb* could not be detected in any of the isolates. Gene *hvgA*, marker of ST-17, was also absent in all isolates belonging to serotype III and the remaining ones. Isolates harbored from four to seven of the assayed virulence genes. The genes *spb1, hylB* and *cylE* were present in all the isolates meanwhile, *bca*, *cpsA*, and *rib* were detected in different frequencies, 36% (20), 96% (54), and 59% (33), respectively (**Table 2**). Pilus typing using three PCR assays, showed that PI-1 and PI-2a genes were absent in all the investigated bovine strains. On the other hand, all isolates harbored the PI-2b gene (**Table 2**).

**Figure 1:**
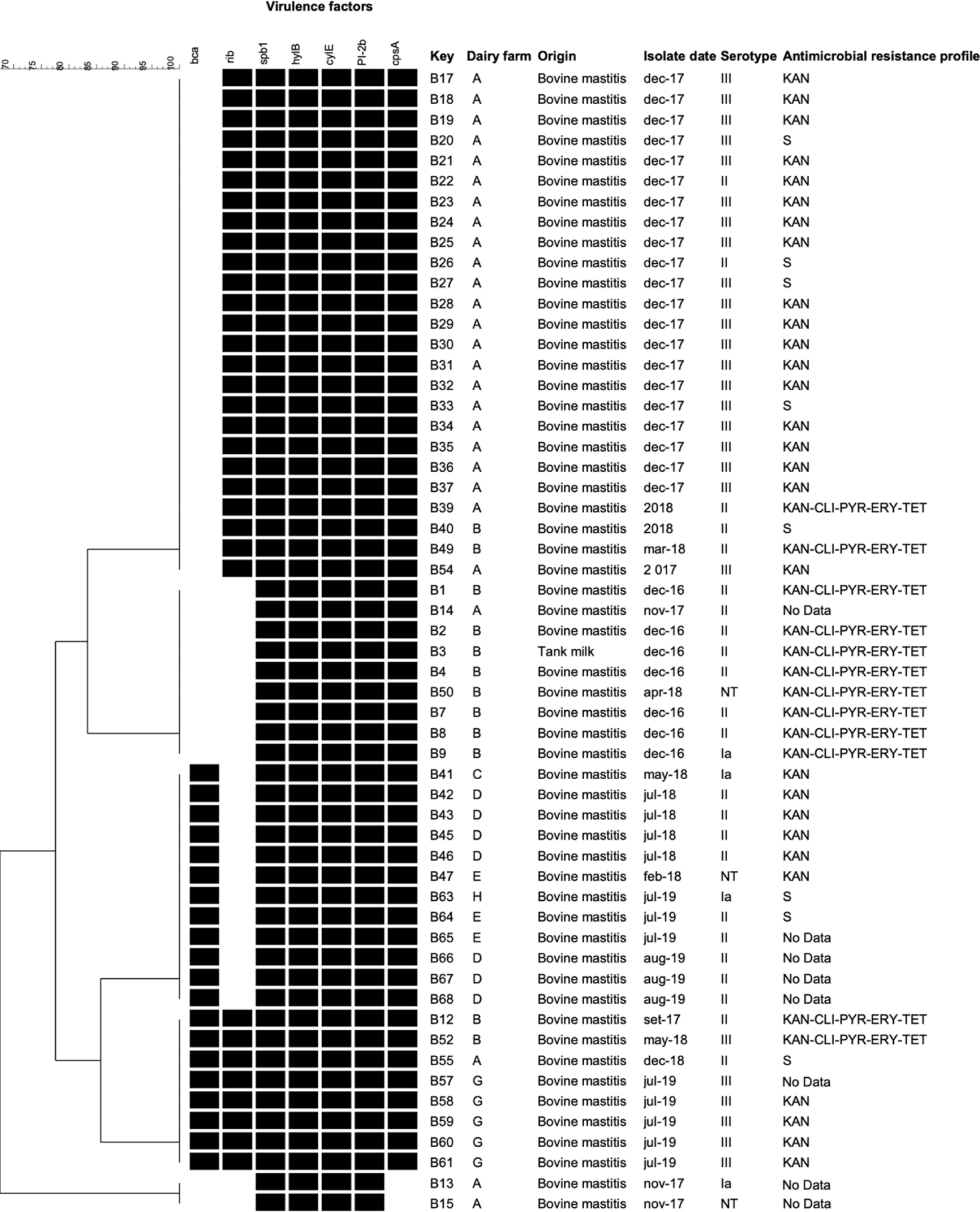
Cluster analysis of *Streptococcus agalactiae* isolated from dairy cattle with mastitis in Argentina based on virulence-associated genes profiles. The presence (black) or absence (white) of genes, the isolate name, dairy farm, origin, isolation date, and serotype of the isolates are shown. The antimicrobial resistance profiles are indicated on the right. NT, non-typeable. Genes not found in any of the studied isolates: *bac, lmb, hvgA, PI, PI-2a*, and *scpB*. S: susceptible to all tested antimicrobial agents.

The cluster analysis taking into account the combinations of the genes detected in the present study showed five virulence profiles shared by isolates from different dairy farm, except one (*spB1*-*hylB*-*cylE*-*PI-2b*) which was presented only by two isolates from dairy farm A. No one of the profiles could be associated with a particular serotype (**Figure 1**).

Regarding antimicrobial resistance, 48 *S. agalactiae* isolates (86%) could be tested using a disc diffusion method. We did not have data on the remaining eight isolates due to problems with contamination during susceptibility testing. In relation to the aminoglycoside kanamycin, since not reference breakpoints values were available, only isolates presenting no inhibition area were considered as resistant. The isolates without inhibition area were 40, representing 83% of the tested isolates. Twelve isolates, eleven of them obtained from the dairy farm B and one from the farm A, were resistance to five antimicrobials, kanamycin, clindamycin, pirlimycin, erythromycin and, tetracycline (**Figure 1**).

To investigate genetic antimicrobial resistance, PCR assays for genes accounting for resistance to several antibiotics were carried out. In relation to tetracycline resistance, the efflux genes *tet*(K) and *tet*(L) and the ribosomal protection genes *tet*(M) and *tet*(O) were amplified. The gene *tet*(O) was recovered in all tetracycline-resistant strains, and, also, in 16 strains which did not present phenotypic susceptibility for tetracycline; the gene *tet(*M), in 5 isolates (B41, B57, B58, B59 and B57).

In relation to erythromycin resistance, all resistant isolates exhibited resistance associated to *ermB* gene indicating the presence of a target-site modification by a ribosomal methylase. Six susceptible strains, also carried that gene. Genes *aphA3* and *aad6*, related to aminoglycosides resistance, were also amplified. The gene *aad6* was not detected meanwhile *aphA3*, related to kanamycin resistance, was detected in 18 isolates. The detection of *lnuB* in clindamycin and pirlimycin-resistant isolates would explain the resistance observed to lincosamides. The positive-isolates were B4, B39, and B49. The average correlation rate between resistant phenotypes and genotypes was 65.62% (**Table 3**). The calculations were made taking into account only those resistant isolates that were studied by both analyses (phenotypic and genetic resistance). Given each of the antimicrobial classes individually, tetracyclines and macrolides showed the highest correlation (100%), while aminoglycosides and lincosamides presented lower correlations (37.5% and 25% respectively). On the other hand, the 50% of strains that presented phenotypic susceptibility for all antimicrobial classes carried some resistance genes.

**Table 3:** The correlations between resistance phenotypes and resistance genes of *S. agalactiae* isolates.

Antimicrobial classes	No. of phenotypes of resistance	No. of strains with phenotype of resistance carrying resistance genes	Correlation rate (%)^1^
**Macrolides**	12	12	100
**Aminoglycosides**	40	15	37.5
**Tetracyclines**	12	12	100
**Lincosamides**	12	3	25
**Average**			65.62

^1^For this calculation, only those resistant isolates studied by both analyses (phenotypic and genetic resistance) were taken into account.

## Discussion

*Streptococcus agalactiae* is considered one of the major mastitis pathogens and, for our knowledge, this is the first molecular study that characterizes *S. agalactiae* isolates circulating among cattle with mastitis in Argentina. Of the 68 original strains identified as *S. agalactiae* by biochemical tests, only 56 ones were confirmed genetically.

Capsular serotyping is a classical method used for epidemiological studies in *S. agalactiae* and ten serotypes are identified based on Cps types. Serotypes III and II were the predominant ones among *S. agalactiae* Argentinean bovine strains (87%), followed by a low frequency of strains classified as Ia and non-typeable. Serotype III, and especially lineage ST-17, is particularly important in human infections because it causes the majority of infections in neonates (Manning, 2014). In this study, we did not detect *hvgA*, a gene encoding an ST-17–specific surface-anchored protein, critical virulence trait of neonatal disease associated-S *agalactiae* (Tazi et al., 2010). The distribution of *S. agalactiae* serotypes involved in bovine mastitis is variable worldwide and certain types appear to predominate within geographical regions. Serotype II was the most prevalent in Canada (Zhao et al., 2006), Eastern-China, USA (together with Ia) (Dogan et al., 2005; Yang et al., 2013) and Germany (together with non-typeable) (Merl et al., 2003). On the other hand, serotypes V and IV were the most prevalent in Norway (Radtke et al., 2012). The dominant serotypes in Poland were Ia and II although also serotypes Ib, III, IV, and V were detected (Kaczorek et al., 2017) while in strains originating from dairy herds in France, non-typeable strains were detected at the highest frequency, followed by serotypes III and IV (Brochet et al., 2006). In Brazil, five capsular types were identified (Ia, Ib, II, III, and IV) (Duarte et al., 2005; Pinto et al., 2014; Carvalho-Castro et al., 2017), being III and II the most prevalent ones, just like our results and, in Iran, only were detected these last two serotypes (Emaneini et al., 2016).

In relation to the virulence genes screened in this study, five virulence profiles were detected, which included *spb1*, *hylB*, *cylE*, and PI-2b. The product of the *spb1* gene has been proposed as a factor implicated in adhesion to epithelial cells (Adderson et al., 2003). The gene *hylB*, encodes an important marker of virulence that degrades hyaluronic acid, facilitating bacterial dissemination (Baker and Pritchard, 2000). Other authors also detected *hylB* in all the studied samples (Carvalho-Castro et al., 2017; Pang et al., 2017). CylE is a pore-forming toxin, involved in tissue injury and the systemic spread of bacteria (Doran et al., 2002; Doran et al., 2003; Reiß et al., 2011). The expression of this β-hemolysin has proapoptotic, pro-inflammatory, and cytotoxic effects (Randis et al., 2014). Previous studies reported the presence of this gene in 78% of Poland strains (Kaczorek et al., 2017) and in 100% of Chinese ones (Pang et al., 2017).

Pilus structures in *S. agalactiae* facilitate colonization and invasion of host tissues and participate in biofilm formation. These structures are encoded in islands (PI) and three of them, PI-1, PI-2a, and PI-2b, have been identified in highly virulent strains (Margarit et al., 2009). *S. agalactiae* strains carry at least one of the three PI and, some studies highlight that strains of different origins usually harbor different pilus variants; while type 2a (PI-2a) is more common in human strains, type 2b (PI-2b) is more frequent in bovine isolates (Martins et al., 2013; Otaguiri et al., 2013; Carvalho-Castro et al., 2017; Pang et al., 2017). Our results agree with previous studies in which all the bovine *S. agalactiae* strains from China carried only PI-2b (Yang et al., 2013; Pang et al., 2017).

The studied isolates differed mainly by the presence of *bca* and *rib*. Someone harbored one of the two genes (*bca*: 20 isolates, 36%; *rib*: 33 isolates, 59%) others both (8 isolates, 14%), and some, neither of them (9 isolates, 16%). These genes belong to the alpha-like surface protein family and are important in the pathogenicity. It was not possible associate the presence/absence of these genes with serotype. The protein ą-C, encoded by the *bca* gene mediates the internalization of the bacteria to host cells (Baron et al., 2004) and Rib is only present in invasive strains (Lindahl et al., 2005). Studies carried out in Poland reported the presence of *bca* in 37% (Kaczorek et al., 2017) and in Brazil it varied between 3% and 79%, depending on the geographical location (Duarte et al., 2004; Carvalho-Castro et al., 2017). For *rib*, previous studies reported the presence in bovine strains of 33% in Poland (Kaczorek et al., 2017) and 89% in Iran (Emaneini et al., 2016).

The isolates were negative for the virulence genes *bac*, *lmb*, and *scpB*. Earlier molecular reports showed that most bovine isolates lack surface proteins-encoding genes *scpB* and *lmb*, in contrast to human isolates (Franken et al., 2001), but that however they can be detected in some *S. agalactiae* bovine strains (Rato et al., 2013).

In order to have a global view of antimicrobial susceptibility occurrence, isolates were tested against eight antimicrobial agents selected taking into account their use for mastitis treatment in cattle (penicillin, oxacillin, kanamycin, pirlimycin, and tetracycline) or/and in human medicine (penicillin, erythromycin, clindamycin, levofloxacin). We evaluated their antimicrobial resistance profiles using also antibiotics of human use considering that this information can assist in critical decision making as part of the concept “One health”. The most commonly used antimicrobial classes for the treatment of streptococcal mastitis are β-lactams and macrolides (Denamiel et al., 2005). On the one hand, we did detect resistance to macrolides (erythromycin and clindamycin), and on the other, besides, resistance to the kanamycin. The practice shows the intensive use in Argentinean dairy farms of a product containing this aminoglycoside in association with a β-lactam drug. The absence of resistance to penicillin and oxacillin observed in this study, and in other ones worldwide (Haenni et al., 2018), indicates that β-lactam antibiotics should remain the drugs of choice in the treatment of streptococcal mastitis. However, the high level of resistance to kanamycin detected predicts lack of synergy when associated with β-lactams leading to therapy failure (Chow, 2000).

According to the World Organization for Animal Health (OIE, 2016), between 2010 and 2015, tetracyclines and macrolides were the two classes of antibiotics most commonly used in animals worldwide. We detected resistance to tetracycline and erythromycin, agreeing with previous reports on bovine strains recovered in Brazil, Poland and, Portugal (Rato et al., 2013; Kaczorek et al., 2017; Tomazi et al., 2018). On the other hand, a previous study carried on milk from infected udders reported erythromycin and clindamycin-resistant Argentinean *Streptococcus* isolates (Denamiel et al., 2005). In addition to resistance to tetracycline, erythromycin, clindamycin, and kanamycin, the multi-drug resistant (MDR) isolates were resistant to pirlimycin, a lincosamide approved only for veterinary use. This antimicrobial agent is available for intramammary administration to treat mastitis caused by gram-positive cocci and pirlimycin resistance among streptococci has been reported previously (Pol and Ruegg, 2007; Tomazi et al., 2018).

In streptococci, resistance to tetracycline is encoded by ribosome protection genes including *tet*(M) and *tet*(O) or by efflux pump genes, *tet*(K), and *tet*(L) (Rubio-López et al., 2012). Resistance to macrolide are due to two common mechanisms, a ribosome methylase, encoded by the *erm* gene and an active efflux pump by a membrane-bound protein, encoded by the *mef* gene. The former concurrently confers high-level resistance to macrolides, as well as to lincosamide and streptogramin B antibiotics (Ko et al., 2004). All studied macrolide-resistant isolates were also resistant to clindamycin and all of them harbored the *ermB* gene.

Resistance to tetracycline was attributed to the presence of *tet*(O), erythromycin resistance to target site modification encoded by the erythromycin ribosome methylase gene *ermB*, pirlimycin/clindamycin resistance to *lnu*(B), a gene encoding a lincosamide inactivating nucleotidyl transferase and, kanamycin resistance to *aphA3*, a gene encoding an aminoglycoside phosphotransferase. The average correlation between resistance phenotypes and resistance genes of *S. agalactiae* was 65.62%. Particularly, the correlation for tetracycline was 100%. Similar findings were reported by da Silva et al. (2017), in *S. agalactiae* strains isolated from Brazilian mastitic cows and by Liu et al. (2017), in *Staphylococcus aureus* strains isolated from raw milk. Although mastitis therapy is commonly initiated before the results of antimicrobial susceptibility tests of the pathogen are known (Guérin-Faublée et al., 2002), the emergence of resistant pathogens (such as the detected in this study) makes essential performing susceptibility tests for the selection of the appropriate chemotherapeutic agents (Minst et al., 2012).

Farm animal disease control often share active substances with human medicines and, excessive use of antibiotics is associated with the risk of the creation of MDR foodborne pathogens (van Duin and Paterson, 2016). Another point to keep in mind when considering antimicrobial resistance of mastitis pathogens is that the interpretive criteria used for categorizing isolates are based on human data for the majority of compounds tested. They may not accurately reflect the efficacy of the drugs in the treatment of bovine mastitis (Rajala-Schultz et al., 2004) and therefore, veterinary-specific breakpoints are necessary (Thomas et al., 2015).

On the other hand, vaccination is one of the strategies most likely to be implemented to prevent GBS infections. Several GBS vaccine candidates are in development, especially for humans (Bianchi-Jassir et al., 2020). Cps and pilus proteins are some of the main targets proposed for the vaccine. In order to guide vaccine development or before of regulating the use of it, it is essential to answer the question about which serotypes are the most implicated in cases of disease in the country and, if possible, identify alternative targets. In this study, we reported that serotypes III and II were the most prevalent ones (87%) and the presence of *spb1*, *hylB*, *cylE*, and PI-2b in all the isolates. These data could be of interest in the perspective of a future vaccine.

## Conclusions

The results of this study are the first molecular data on *S. agalactiae* isolates causing bovine mastitis in Argentina. We detected several virulence and antimicrobial susceptibility profiles associated with *S. agalactiae* intramammary infections. On the one hand, we found the all the isolates harbored the genes *spb1*, *hylB*, *cylE*, and PI-2b, and the predominance of serotype III and II. On the other hand, we detected strains MDR to clinical and veterinary relevant antimicrobials, and, several resistance-encoding genes. These data present us with the future challenge of closely monitoring the spread of MDR strains, to explore the molecular mechanisms responsible for the antimicrobial resistance, and provide a foundation for the development of diagnostic, prophylactic, and therapeutic methods, including the perspective of a vaccine.

## Data Availability Statement

The raw data supporting the conclusions of this article will be made available by the authors, without undue reservation.

## Author Contributions

Conceptualization and design of the study, AS and AB. Methodology, LH, EB, JC, CC, CM, and AB. Data analysis and interpretation, LH, JC, AB, and AS. Writing original draft preparation, AS and LH. Critical revisions and writing of the revised manuscript, AS. Supervision and administration project, AS. All authors contributed to the article and approved the submitted version.

## Funding

This research was funded by Fondo para la Investigación Científica y Tecnológica (PICT 1139-17) and Consejo Nacional de Investigaciones Científicas y Técnicas (PIP 365/15, PUE CIVETAN).

## Conflict of Interest

The authors declare that the research was conducted in the absence of any commercial or financial relationships that could be construed as a potential conflict of interest.
