# Supplementary material for: Multidrug Resistance and Molecular Characterization of Streptococcus agalactiae Isolates From Dairy Cattle With Mastitis
Source: Front Cell Infect Microbiol. 2021 Apr 30;11:647324. doi: 10.3389/fcimb.2021.647324 (PMC8120232; doi:10.3389/fcimb.2021.647324)
Supplement: Supplementary file 1 [file Table_1.docx]

Supplementary Material

**Table S1.** Antibiotics tested for *Streptococcus agalactiae* isolates from bovine mastitis and the used breakpoints values. S, susceptible; R, resistant (according to the diameters of the inhibition area).

| **Antimicrobial** | **Concentration (μg/disc)** | **S** | **R** | **Reference** |
| --- | --- | --- | --- | --- |
| **Aminoglycosides** |  |  |  |  |
| •Kanamycin ^1^ | 120 |  |  | Without reference |
| **Lincosamides** |  |  |  |  |
| •Clindamycin | 2 | ≥19 | ≤15 | Human *Streptococcus* beta-hemolytic (CLSI, 2019) |
| •Pirlimycin | 2 | ≥13 | ≤12 | Bovine *Streptococcus* beta-hemolytic (CLSI, 2018) |
| **Macrolides** |  |  |  |  |
| •Erythromycin | 15 | ≥21 | ≤15 | Human *Streptococcus* beta-hemolytic (CLSI, 2019) |
| **Quinolones** |  |  |  |  |
| Levofloxacin | 5 | ≥17 | ≤13 | Human *Streptococcus* beta-hemolytic (CLSI, 2019) |
| **Tetracyclines** |  |  |  |  |
| •Tetracycline | 30 | ≥23 | ≤18 | Human *Streptococcus* beta-hemolytic (CLSI, 2019) |
| **Beta-lactams** |  |  |  |  |
| Penicillin | 10 U | ≥24 |  | Human *Streptococcus* beta-hemolytic (CLSI, 2019) |
| Oxacillin | 1 | ≥20 | ≤12 | Human *S. pneumoniae* (CLSI, 2019) |

• Resistance was detected. ^1^ Only isolates presenting no zones of inhibition were considered as resistant.

**References**

CLSI - Clinical and Laboratory Standards Institute (2018). *Performance Standards for Antimicrobial* *Disk and Dilution Susceptibility Tests for Bacteria Isolated from Animals*. 4th ed., ed. Clinical and Laboratory Standards Institute Wayne, PA, USA.

CLSI - Clinical and Laboratory Standards Institute (2019). *Performance Standards for Antimicrobial Susceptibility Testing*. 29th ed., ed. Clinical and Laboratory Standards Institute Wayne, PA, USA.
